# Supplementary material for: The closed eye harbours a unique microbiome in dry eye disease
Source: Sci Rep. 2020 Jul 21;10:12035. doi: 10.1038/s41598-020-68952-w (PMC7374690; doi:10.1038/s41598-020-68952-w)
Supplement: Supplementary file 1 — Supplementary information [file 41598_2020_68952_MOESM1_ESM.pdf]

Supplement

The closed eye harbors a unique microbiome in dry eye disease

Kent A. Willis, **Cameron K. Postnikoff**, **Gabriel Rezonzew**, **Kelly Nichols**, **Amit Gaggar**, **Charitharth V. Lal**

Supplemental Fig S1.

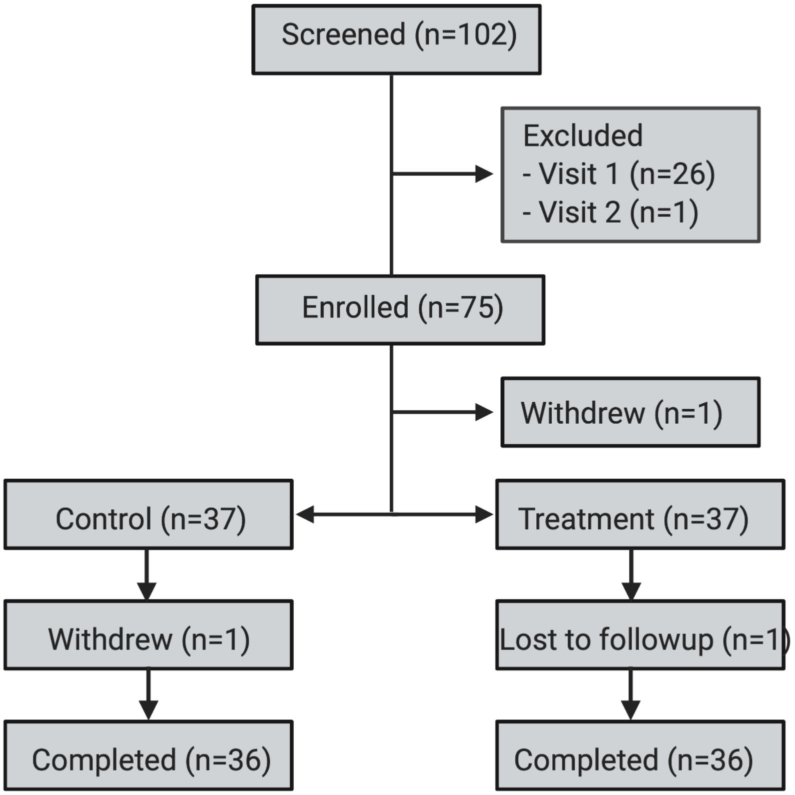

Fig. S1. Consolidated standards of reporting trials subject allocation diagram. CONSORT subject allocation diagram. Figure generated by BioRender.

Supplemental Fig S2.

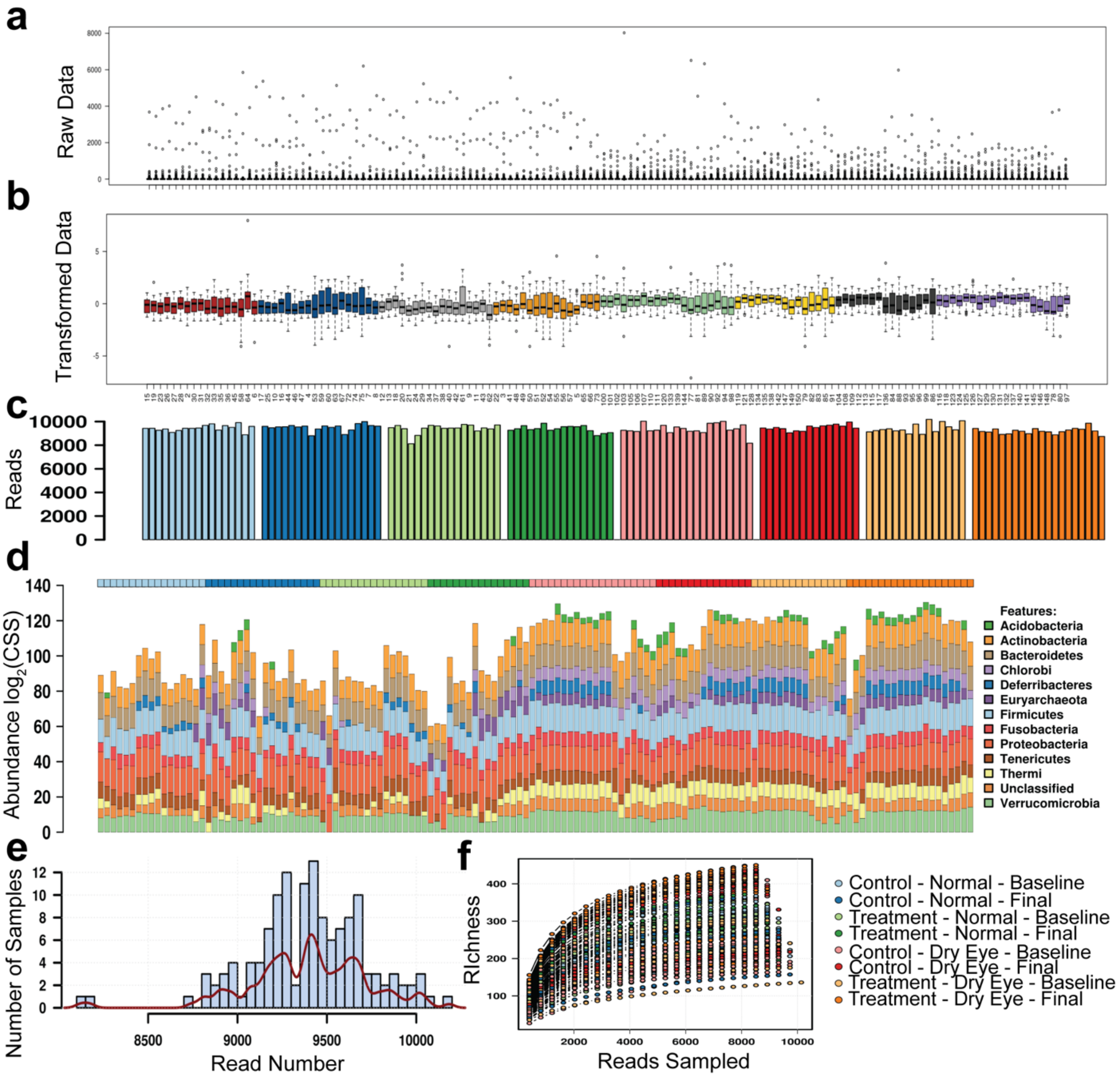

Fig. S2. Read depth and quality are consistent across clinical groupings.
(a) Raw sequencing data.
(b) Log_2-transformed relative abundance.
(c) Sequencing depth.
(d) Relative abundance at the phylum level.
(e) Sequence reads per sample.
(f) Read depth.

Supplemental Fig. S3.

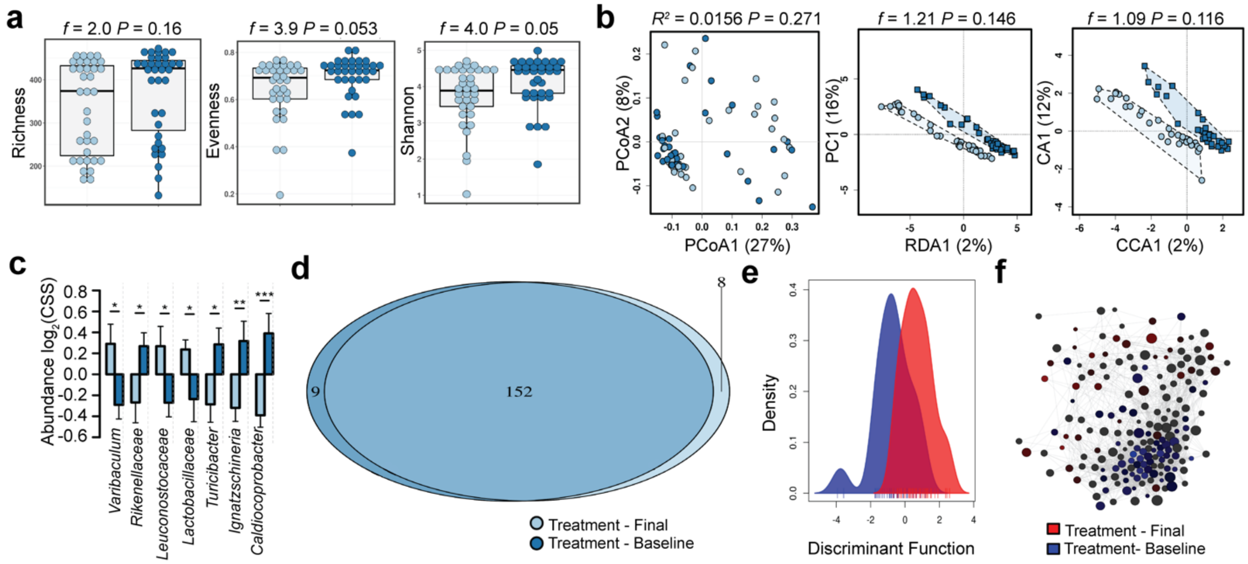

Fig. S3. Limited changes occur in the dry eye microbiome with daily eye rinse.
(a) Alpha diversity.
(b) Beta diversity.
(c) Relative abundance of bacterial genera. Two-way ANOVA, * *P* =0.05, ** *P* =0.01, *** *P* = 0.001.
(d) Core microbiome analysis.
(e) Discriminant analysis of principal components.
(f) Spearman network analysis at the genus level.

Supplemental Fig. S4

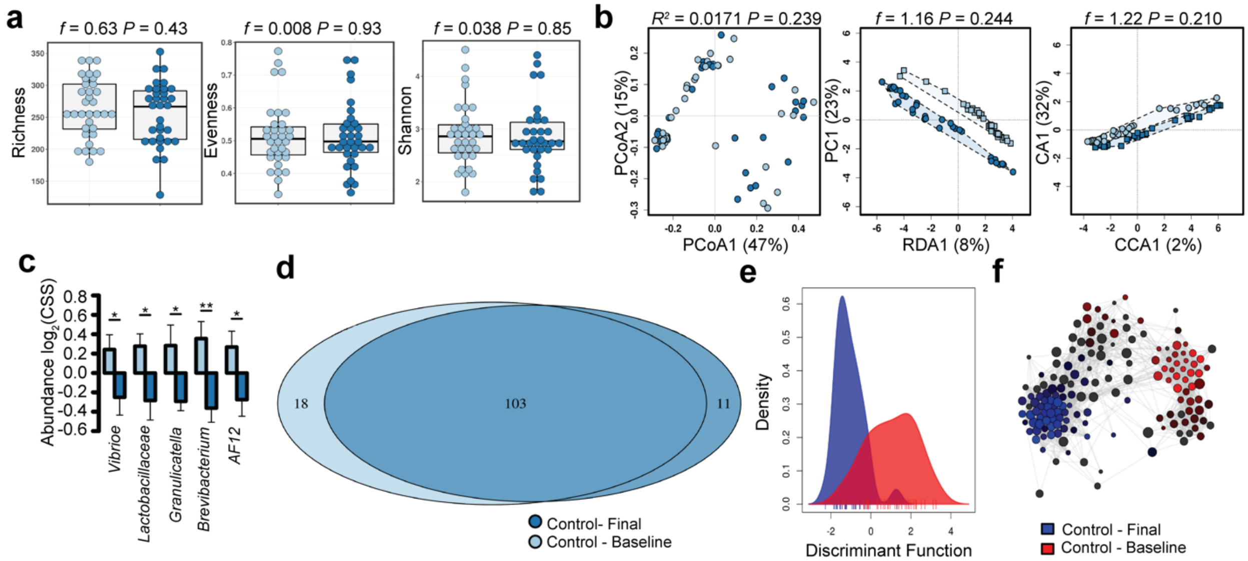

Fig. S4. Limited changes occur in the normal eye microbiome with eye rinse.
(a) Alpha diversity.
(b) Beta diversity.
(c) Relative abundance of bacterial orders. Two-way ANOVA, * *P* =0.05, ** *P* =0.01, *** *P* = 0.001.
(d) Core microbiome analysis.
(e) Discriminant analysis of principal components.
(f) Spearman network analysis at the genus level.

Supplemental Fig. S5.

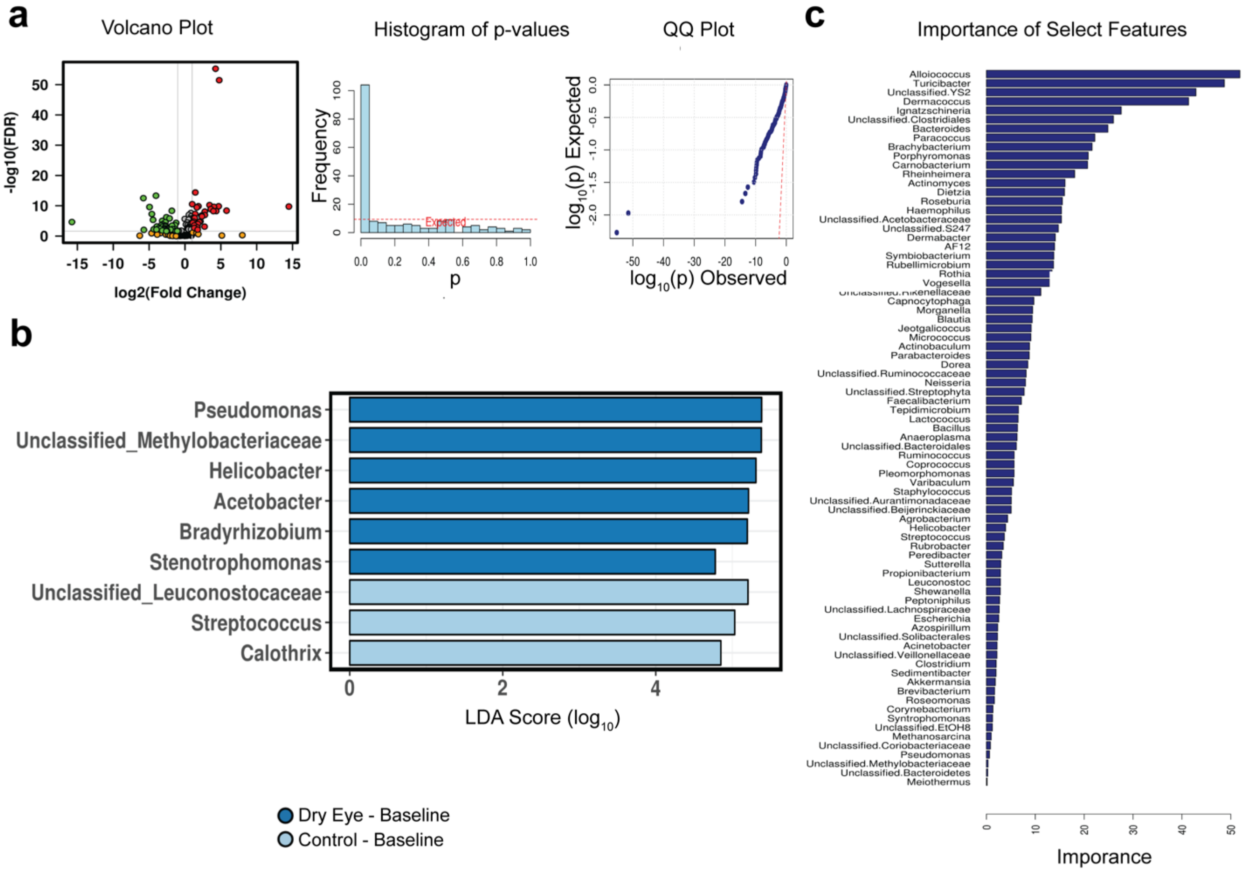

Fig. S5. The acquisition of unique taxa separates the dry from the normal eye at baseline.
(a) Negative binomial distribution (DESeq2 function).
(b) Linear discriminant analysis of effect size (LEfSe).
(c) Variable importance analysis of random forest classifier.

Supplemental Fig. S6.

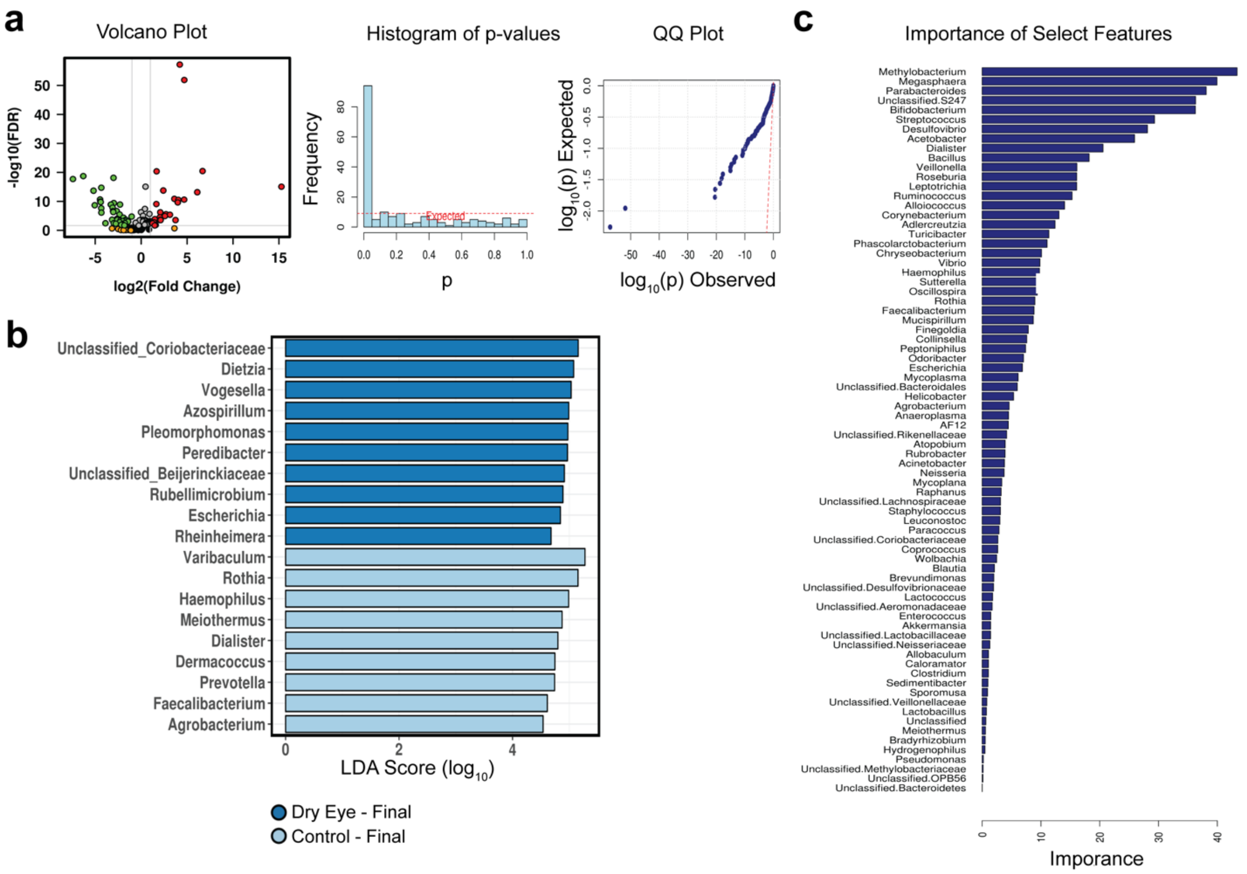

Fig. S6. Additional unique taxa separate the dry from the normal eye after a month.
(a) Negative binomial distribution (DESeq2 function).
(b) Linear discriminant analysis of effect size (LEfSe).
(c) Variable importance analysis of random forest classifier.

Supplemental Table 1.

	**Normal**		**Dry Eye**	
	Normal	Mild	Moderate	Severe
**DEQ5**	≤ 5	≤ 5	≥ 6	≥ 12
	All of the below:		At least one of the following:	
**Phenol Red Thread, mm**	> 10, bilateral	> 10, bilateral	< 10, one eye	< 10, one eye
**NIKBUT, s**	> 5, bilateral	> 5, bilateral	< 10, one eye	< 5, one eye
**InflammaDry**	Negative, both eyes	Positive, one or both eyes	Positive, one or both eyes	Positive, one or both eyes

Supplemental Table 2.

Taxa (genus)	P (biological condition)	Adjusted P (Bonferroni)	FDR
Unclassified.OPB56	< 1E-22	< 1E-22	< 1E-22
Unclassified.Methylobacteriaceae	< 1E-22	1E-21	2.00E-22
Unclassified.Bacteroidetes	< 1E-22	< 1E-22	< 1E-22
Pseudomonas	< 1E-22	< 1E-22	< 1E-22
Meiothermus	< 1E-22	< 1E-22	< 1E-22
Hydrogenophilus	2.5E-19	4.6E-17	7.7E-18
Unclassified.Chitinophagaceae	7.6E-19	1.4E-16	2E-17
Bradyrhizobium	6E-18	1.1E-15	1.4E-16
Unclassified	3.6E-16	6.6E-14	7.4E-15
Unclassified.Aeromonadaceae	7.2E-16	1.3E-13	1.3E-14
Raphanus	2.7E-15	5E-13	4.5E-14
Unclassified.Coriobacteriaceae	1.4E-14	2.6E-12	2.1E-13
Unclassified.Desulfovibrionaceae	4.8E-13	8.8E-11	6.8E-12
Brevundimonas	5.8E-12	1.1E-09	7.6E-11
Lactobacillus	5.4E-11	9.9E-09	6.6E-10
Leuconostoc	1.7E-10	0.000000031	0.000000002
Allobaculum	5.9E-10	0.00000011	6.4E-09
Unclassified.Beijerinckiaceae	1.1E-09	0.0000002	0.000000011
Unclassified.Aurantimonadaceae	1.4E-09	0.00000026	0.000000014
Shewanella	1.8E-09	0.00000033	0.000000015
Corynebacterium	1.8E-09	0.00000033	0.000000015
Acinetobacter	1.8E-09	0.00000033	0.000000015
Roseomonas	2.2E-09	0.0000004	0.000000018
Phenylobacterium	4.7E-09	0.00000086	0.000000036
Azospirillum	5.8E-09	0.0000011	0.000000043
Aerococcus	0.000000025	0.0000046	0.00000018
Lysobacter	0.000000043	0.0000079	0.00000029
Pleomorphomonas	0.000000063	0.000012	0.00000041
Brevibacterium	0.000000068	0.000013	0.00000043
Morganella	0.000000094	0.000017	0.00000058
Symbiobacterium	0.000000097	0.000018	0.00000058
Peredibacter	0.0000001	0.000018	0.00000058
Bacillus	0.00000012	0.000022	0.00000067
Unclassified.Solibacterales	0.00000024	0.000044	0.0000013
Jeotgalicoccus	0.00000031	0.000057	0.0000016
Sedimentibacter	0.00000088	0.00016	0.0000045
Caloramator	0.000001	0.00018	0.000005

Wolbachia	0.0000012	0.00022	0.0000058
Unclassified.Caulobacteraceae	0.0000013	0.00024	0.0000061
Streptococcus	0.0000022	0.0004	0.00001
Unclassified.Veillonellaceae	0.0000024	0.00044	0.000011
Unclassified.Neisseriaceae	0.0000041	0.00075	0.000018
Mycoplana	0.0000043	0.00079	0.000018
Mucispirillum	0.0000046	0.00085	0.000019
Sporomusa	0.0000056	0.001	0.000023
Caldicoprobacter	0.0000077	0.0014	0.000031
Caldinitratiruptor	0.000013	0.0024	0.000051
Akkermansia	0.000015	0.0028	0.000058
Anaerococcus	0.000018	0.0033	0.000068
Leptotrichia	0.000024	0.0044	0.000088
Devosia	0.000025	0.0046	0.00009
Unclassified.Halanaerobiales	0.000027	0.005	0.000096
Neisseria	0.000032	0.0059	0.00011
Balneimonas	0.000033	0.0061	0.00011
Unclassified.Lactobacillaceae	0.000038	0.007	0.00013
Methanosarcina	0.000045	0.0083	0.00015
Desulfosporosinus	0.000048	0.0088	0.00015
Dietzia	0.000087	0.016	0.00028
Lactococcus	0.000093	0.017	0.00029
Unclassified.MLE112	0.00012	0.022	0.00037
Ruminococcus	0.00015	0.028	0.00045
Gordonia	0.00015	0.028	0.00045
Mycoplasma	0.00018	0.033	0.00053
Sutterella	0.00019	0.035	0.00055
Paenibacillus	0.00025	0.046	0.0007
Finegoldia	0.00025	0.046	0.0007
Unclassified.Paenibacillaceae	0.00027	0.05	0.00074
Rubrobacter	0.00032	0.059	0.00087
Helicobacter	0.0004	0.074	0.0011
Unclassified.EtOH8	0.00046	0.085	0.0012
Actinobaculum	0.00056	0.1	0.0015
Micrococcus	0.00063	0.12	0.0016
Lutispora	0.00078	0.14	0.002
Brachybacterium	0.00088	0.16	0.0022
Sphingobium	0.0011	0.2	0.0027
Vogesella	0.0012	0.22	0.0029
Unclassified.Lachnospiraceae	0.0013	0.24	0.003

Unclassified.Clostridiales	0.0013	0.24	0.003
Staphylococcus	0.0013	0.24	0.003
Moraxella	0.0013	0.24	0.003
Propionibacterium	0.0015	0.28	0.0034
Unclassified.Bacillales	0.0017	0.31	0.0038
Agrobacterium	0.0018	0.33	0.004
Rothia	0.002	0.37	0.0044
Unclassified.Barnesiellaceae	0.0021	0.39	0.0045
Unclassified.Acetobacteraceae	0.0021	0.39	0.0045
Alloiococcus	0.0026	0.48	0.0055
Unclassified.Aerococcaceae	0.0029	0.53	0.0061
Peptostreptococcus	0.0034	0.63	0.007
Escherichia	0.0037	0.68	0.0076
Coprococcus	0.0048	0.88	0.0096
Adlercreutzia	0.0048	0.88	0.0096
Oscillospira	0.0052	0.96	0.01
Comamonas	0.0063	1	0.012
Clostridium	0.0067	1	0.013
Unclassified.ML615J28	0.0068	1	0.013
Unclassified.Rikenellaceae	0.0083	1	0.016
Syntrophomonas	0.009	1	0.017
Unclassified.Ruminococcaceae	0.01	1	0.019
SMB53	0.011	1	0.02
Vibrio	0.012	1	0.022
Paraprevotella	0.012	1	0.022
Bacteroides	0.014	1	0.025
Unclassified.S247	0.016	1	0.028
Pantoea	0.016	1	0.028
Paracoccus	0.018	1	0.031
Haemophilus	0.018	1	0.031
Unclassified.03196G20	0.02	1	0.034
Rubellimicrobium	0.021	1	0.035
Peptoniphilus	0.022	1	0.037
Blautia	0.023	1	0.038
Candidatus_Arthromitus	0.028	1	0.046
Citrobacter	0.034	1	0.055
Veillonella	0.035	1	0.056
Capnocytophaga	0.036	1	0.058
Parabacteroides	0.037	1	0.058
Lachnospira	0.037	1	0.058

Roseburia	0.04	1	0.062
Anaeroplasma	0.047	1	0.073
Unclassified.Streptophyta	0.049	1	0.074
Unclassified.Gemellaceae	0.049	1	0.074
Enterococcus	0.049	1	0.074
Turicibacter	0.05	1	0.074
AF12	0.05	1	0.074
Collinsella	0.059	1	0.086
Chryseobacterium	0.059	1	0.086
Eubacterium	0.063	1	0.091
Kocuria	0.067	1	0.096
Desulfovibrio	0.073	1	0.1
Pelosinus	0.079	1	0.11
Faecalibacterium	0.08	1	0.11
Eikenella	0.08	1	0.11
Phascolarctobacterium	0.087	1	0.12
WAL_1855D	0.096	1	0.13
Arthrobacter	0.11	1	0.15
Shuttleworthia	0.13	1	0.17
Selenomonas	0.13	1	0.17
Actinomyces	0.13	1	0.17
Bifidobacterium	0.14	1	0.19
Lautropia	0.15	1	0.2
Unclassified.RF32	0.16	1	0.21
Sneathia	0.16	1	0.21
Phyllobacterium	0.17	1	0.22
Granulicatella	0.17	1	0.22
Varibaculum	0.18	1	0.23
Abiotrophia	0.18	1	0.23
Unclassified.Erysipelotrichaceae	0.19	1	0.24
Dorea	0.19	1	0.24
Dermabacter	0.2	1	0.25
X168	0.24	1	0.29
Unclassified.Bacteroidales	0.24	1	0.29
Achromobacter	0.26	1	0.31
Geobacter	0.28	1	0.34
Calothrix	0.32	1	0.38
Megasphaera	0.33	1	0.39
Aggregatibacter	0.34	1	0.4
Rheinheimera	0.39	1	0.45

Facklamia	0.39	1	0.45
Acetobacter	0.39	1	0.45
Dermacoccus	0.43	1	0.49
Stenotrophomonas	0.46	1	0.53
Unclassified.YS2	0.47	1	0.53
Enhydrobacter	0.47	1	0.53
Odoribacter	0.49	1	0.55
Dehalobacterium	0.49	1	0.55
Anaerostipes	0.54	1	0.6
Carnobacterium	0.56	1	0.62
Porphyromonas	0.58	1	0.63
Bilophila	0.58	1	0.63
rc44	0.59	1	0.64
Salinispora	0.61	1	0.65
Chroococcidiopsis	0.61	1	0.65
Prevotella	0.62	1	0.66
Atopobium	0.64	1	0.68
Fusobacterium	0.68	1	0.71
Campylobacter	0.69	1	0.72
Acidaminococcus	0.75	1	0.78
Methylobacterium	0.79	1	0.82
Mycobacterium	0.89	1	0.91
Proteus	0.9	1	0.92
Sphingomonas	0.91	1	0.93
Janthinobacterium	0.92	1	0.93
Dialister	0.93	1	0.94
Tissierella_Soehngenia	0.95	1	0.95

Supplemental Table 3.

Taxa	P Assignment	FDR Assignment	P adjusted Assignment
Unclassified.OPB56	< 1E-22	< 1E-22	< 1E-22
Unclassified.Bacteroidetes	< 1E-22	< 1E-22	< 1E-22
Pseudomonas	< 1E-22	< 1E-22	1E-22
Meiothermus	< 1E-22	2E-22	9E-22
Unclassified.Methylobacteriaceae	2E-22	6.3E-21	3.1E-20
Hydrogenophilus	5.8E-18	1.8E-16	1E-15
Unclassified.Chitinophagaceae	7.1E-18	1.9E-16	1.3E-15
Bradyrhizobium	1.4E-16	3.2E-15	2.5E-14
Unclassified	7.9E-15	1.6E-13	1.4E-12

Unclassified.Aeromonadaceae	1.1E-14	2E-13	1.9E-12
Raphanus	6.4E-14	1.1E-12	1.1E-11
Unclassified.Coriobacteriaceae	2.4E-13	3.7E-12	4.2E-11
Unclassified.Desulfovibrionaceae	8.6E-12	1.2E-10	1.5E-09
Brevundimonas	1.2E-10	1.6E-09	0.000000021
Lactobacillus	4.7E-10	5.8E-09	0.00000008
Unclassified.Aurantimonadaceae	0.000000002	0.000000023	0.00000034
Allobaculum	2.9E-09	0.000000031	0.00000049
Leuconostoc	3.1E-09	0.000000032	0.00000052
Unclassified.Beijerinckiaceae	0.000000009	0.000000087	0.0000015
Corynebacterium	0.000000021	0.00000019	0.0000035
Shewanella	0.000000023	0.0000002	0.0000038
Acinetobacter	0.000000028	0.00000023	0.0000046
Azospirillum	0.000000057	0.00000046	0.0000092
Phenylobacterium	0.000000068	0.00000052	0.000011
Roseomonas	0.00000012	0.00000088	0.000019
Bacillus	0.00000023	0.0000016	0.000037
Lysobacter	0.00000025	0.0000017	0.00004
Aerococcus	0.00000033	0.0000022	0.000052
Pleomorphomonas	0.0000006	0.0000038	0.000094
Morganella	0.00000094	0.0000058	0.00015
Peredibacter	0.00000099	0.0000059	0.00015
Brevibacterium	0.0000022	0.000013	0.00034
Symbiobacterium	0.0000024	0.000013	0.00036
Unclassified.Solibacterales	0.0000033	0.000018	0.0005
Unclassified.Caulobacteraceae	0.0000096	0.00005	0.0014
Wolbachia	0.000014	0.00007	0.0021
Streptococcus	0.000014	0.00007	0.0021
Akkermansia	0.000018	0.000087	0.0026
Mycoplana	0.000024	0.00011	0.0035
Jeotgalicoccus	0.000033	0.00015	0.0048
Mucispirillum	0.000051	0.00023	0.0073
Unclassified.Neisseriaceae	0.000069	0.0003	0.0099
Caldinitratiruptor	0.00012	0.00051	0.017
Leptotrichia	0.00018	0.00075	0.025
Unclassified.Lactobacillaceae	0.00019	0.00076	0.027
Balneimonas	0.00019	0.00076	0.027
Devosia	0.00024	0.00094	0.033
Neisseria	0.00029	0.0011	0.04
Ruminococcus	0.00056	0.0021	0.076

Lactococcus	0.0008	0.0029	0.11
Dietzia	0.0008	0.0029	0.11
Anaerococcus	0.00089	0.0031	0.12
Sutterella	0.0014	0.0049	0.18
Mycoplasma	0.0019	0.0064	0.25
Finegoldia	0.0019	0.0064	0.25
Unclassified.Veillonellaceae	0.002	0.0066	0.26
Paenibacillus	0.0023	0.0073	0.29
Caloramator	0.0023	0.0073	0.29
Gordonia	0.0024	0.0075	0.3
Rubrobacter	0.0026	0.008	0.32
Unclassified.Halanaerobiales	0.0038	0.011	0.47
Caldicoprobacter	0.0038	0.011	0.47
Helicobacter	0.004	0.012	0.49
Actinobaculum	0.0044	0.013	0.53
Micrococcus	0.0048	0.014	0.58
Sporomusa	0.0052	0.014	0.62
Brachybacterium	0.0076	0.021	0.9
Sedimentibacter	0.0082	0.022	0.96
Unclassified.Lachnospiraceae	0.0084	0.022	0.97
Unclassified.MLE112	0.0089	0.023	1
Staphylococcus	0.0096	0.025	1
Vogesella	0.0099	0.025	1
Desulfosporosinus	0.01	0.025	1
Unclassified.Clostridiales	0.011	0.027	1
Unclassified.Acetobacteraceae	0.012	0.029	1
Unclassified.Aerococcaceae	0.013	0.031	1
Propionibacterium	0.014	0.033	1
Agrobacterium	0.014	0.033	1
Unclassified.Paenibacillaceae	0.015	0.034	1
Rothia	0.015	0.034	1
Moraxella	0.015	0.034	1
Unclassified.Bacillales	0.018	0.04	1
Alloiococcus	0.018	0.04	1
Peptostreptococcus	0.025	0.055	1
Escherichia	0.028	0.061	1
Coprococcus	0.03	0.064	1
Adlercreutzia	0.031	0.066	1
Unclassified.Barnesiellaceae	0.032	0.067	1
Sphingobium	0.041	0.084	1

Comamonas	0.041	0.084	1
Unclassified.EtOH8	0.046	0.092	1
Bacteroides	0.046	0.092	1
Oscillospira	0.049	0.097	1
Vibrio	0.05	0.098	1
Unclassified.Rikenellaceae	0.055	0.11	1
Methanosarcina	0.06	0.12	1
Unclassified.Ruminococcaceae	0.065	0.12	1
Unclassified.S247	0.073	0.14	1
Blautia	0.083	0.15	1
Lutispora	0.098	0.18	1
Veillonella	0.1	0.18	1
SMB53	0.1	0.18	1
Haemophilus	0.1	0.18	1
Rubellimicrobium	0.11	0.19	1
Paraprevotella	0.11	0.19	1
Unclassified.ML615J28	0.12	0.2	1
Paracoccus	0.12	0.2	1
Anaeroplasma	0.12	0.2	1
Candidatus_Arthromitus	0.14	0.24	1
Pantoea	0.15	0.25	1
Faecalibacterium	0.15	0.25	1
Peptoniphilus	0.16	0.26	1
Parabacteroides	0.18	0.29	1
Syntrophomonas	0.19	0.31	1
Lachnospira	0.2	0.32	1
Capnocytophaga	0.2	0.32	1
Turicibacter	0.21	0.33	1
Roseburia	0.22	0.34	1
Unclassified.Bacteroidales	0.23	0.35	1
Phascolarctobacterium	0.23	0.35	1
Unclassified.Gemellaceae	0.24	0.36	1
Collinsella	0.25	0.38	1
Eubacterium	0.28	0.41	1
Chryseobacterium	0.28	0.41	1
AF12	0.28	0.41	1
Unclassified.Streptophyta	0.29	0.42	1
Unclassified.03196G20	0.3	0.43	1
Dorea	0.32	0.46	1
Desulfovibrio	0.37	0.53	1

WAL_1855D	0.4	0.57	1
Enterococcus	0.44	0.61	1
Eikenella	0.44	0.61	1
Bifidobacterium	0.44	0.61	1
Citrobacter	0.45	0.62	1
Kocuria	0.48	0.65	1
Shuttleworthia	0.49	0.65	1
Clostridium	0.49	0.65	1
Arthrobacter	0.49	0.65	1
Actinomyces	0.55	0.73	1
Selenomonas	0.57	0.74	1
Lautropia	0.57	0.74	1
Unclassified.Erysipelotrichaceae	0.58	0.75	1
Prevotella	0.58	0.75	1
Sneathia	0.59	0.75	1
Unclassified.RF32	0.6	0.76	1
Varibaculum	0.62	0.78	1
Granulicatella	0.62	0.78	1
Abiotrophia	0.64	0.8	1
Pelosinus	0.67	0.83	1
Dermabacter	0.76	0.93	1
Achromobacter	0.8	0.97	1
Calothrix	0.84	1	1
Geobacter	0.85	1	1
Rheinheimera	0.87	1	1
Megasphaera	0.87	1	1
Bilophila	0.9	1	1
X168	0.91	1	1
Acetobacter	0.92	1	1
Dermacoccus	0.93	1	1
Stenotrophomonas	0.94	1	1
rc44	0.95	1	1
Unclassified.YS2	0.97	1	1
Odoribacter	0.97	1	1
Facklamia	0.97	1	1
Enhydrobacter	0.97	1	1
Phyllobacterium	0.98	1	1
Dehalobacterium	0.98	1	1
Tissierella_Soehngenia	1	1	1
Sphingomonas	1	1	1

Salinispora	1	1	1
Proteus	1	1	1
Porphyromonas	1	1	1
Mycobacterium	1	1	1
Methylobacterium	1	1	1
Janthinobacterium	1	1	1
Fusobacterium	1	1	1
Dialister	1	1	1
Chroococcidiopsis	1	1	1
Carnobacterium	1	1	1
Campylobacter	1	1	1
Atopobium	1	1	1
Anaerostipes	1	1	1
Aggregatibacter	1	1	1
Acidaminococcus	1	1	1

Supplemental Table 4.

Taxa (Genus)	Fold Change	P (DESeq2)	Adjusted P (Bonferroni)	FDR
Unclassified.Methylobacteriaceae	25.81	< 1E-22	< 1E-22	< 1E-22
Pseudomonas	18.38	< 1E-22	< 1E-22	< 1E-22
Azospirillum	102.9	3.5E-21	6.3E-19	1.9E-19
Bradyrhizobium	3.2	4.3E-21	7.8E-19	1.9E-19
Unclassified.Coriobacteriaceae	-78.88	1.9E-19	3.5E-17	7E-18
Unclassified.Bacteroidetes	-8.057	7.7E-19	1.4E-16	2.3E-17
Unclassified	1.389	8.8E-16	1.6E-13	1.8E-14
Unclassified.Beijerinckiaceae	39600	9E-16	1.6E-13	1.8E-14
Unclassified.Aeromonadaceae	-20.23	2E-15	3.7E-13	3.7E-14
Agrobacterium	5.308	1.8E-14	3.3E-12	3E-13
Unclassified.Desulfovibrionaceae	-36.35	2.1E-14	3.8E-12	3.2E-13
Phenylobacterium	69	7E-14	1.3E-11	9.8E-13
Lysobacter	12.23	1.3E-11	2.4E-09	1.7E-10
Allobaculum	-22.21	2.2E-11	3.9E-09	2.6E-10
Peredibacter	25.57	2.5E-11	4.5E-09	2.8E-10
Unclassified.Aurantimonadaceae	16.52	2.9E-11	5.3E-09	3.1E-10
Roseomonas	-21.81	1.1E-10	0.000000021	1.2E-09
Mucispirillum	-21.21	1.7E-10	0.000000031	1.6E-09
Unclassified.Caulobacteraceae	15.83	2.3E-10	0.000000042	2.1E-09
Akkermansia	-7.868	3.9E-10	0.00000007	3.3E-09

Mycoplana	3.14	8.5E-10	0.00000015	0.000000007
Meiothermus	-9.829	1.3E-09	0.00000023	9.7E-09
Corynebacterium	-4.932	1.3E-09	0.00000023	9.7E-09
Brevundimonas	-33.23	2.2E-09	0.0000004	0.000000016
Brevibacterium	-8.647	0.000000014	0.0000026	0.0000001
Unclassified.Chitinophagaceae	-20.34	0.000000024	0.0000044	0.00000016
Escherichia	1.325	0.000000041	0.0000074	0.00000026
Hydrogenophilus	-6.369	0.00000022	0.00004	0.0000014
Unclassified.OPB56	-8.359	0.00000024	0.000043	0.0000014
Leuconostoc	-8.183	0.0000005	0.000091	0.0000029
Unclassified.Lachnospiraceae	-1.012	0.00000058	0.0001	0.0000032
Methanosarcina	4.233	0.00000059	0.00011	0.0000032
Shewanella	-20.13	0.00000079	0.00014	0.0000042
Clostridium	1.871	0.0000023	0.00042	0.000012
Unclassified.EtOH8	6.491	0.0000027	0.00049	0.000014
Morganella	-5.069	0.0000036	0.00065	0.000018
Pleomorphomonas	8.764	0.0000044	0.00079	0.000021
Ruminococcus	1.151	0.0000097	0.0018	0.000045
Balneimonas	6.021	0.000012	0.0022	0.000054
Vibrio	-2.046	0.000018	0.0033	0.000081
Lutispora	4.099	0.000033	0.0061	0.00014
Anaerococcus	-3.812	0.000045	0.0081	0.00019
Jeotgalicoccus	-6.116	0.0001	0.019	0.00043
Paraprevotella	2.8	0.00014	0.026	0.00058
Aerococcus	-8.985	0.00023	0.042	0.0009
Vogesella	4.328	0.00024	0.043	0.0009
Propionibacterium	-3.183	0.00024	0.043	0.0009
Rubrobacter	-6.067	0.00025	0.046	0.00093
Coprococcus	1.133	0.00026	0.046	0.00093
Unclassified.Halanaerobiales	4.103	0.00029	0.053	0.001
Unclassified.Acetobacteraceae	13.38	0.0003	0.054	0.001
Rubellimicrobium	4.332	0.0003	0.054	0.001
Unclassified.Ruminococcaceae	1.386	0.00031	0.056	0.001
Gordonia	-1.118	0.00034	0.061	0.0011
Rheinheimera	1.18	0.00036	0.065	0.0012
Lactobacillus	-4.775	0.00046	0.083	0.0015
Lactococcus	-3.43	0.00056	0.1	0.0018
Dorea	1.36	0.00062	0.11	0.0019
Sporomusa	1.93	0.00064	0.12	0.0019
Dietzia	-4.13	0.00068	0.12	0.002

Citrobacter	1.767	0.00094	0.17	0.0027
Streptococcus	-2.691	0.001	0.18	0.0029
Anaeroplasma	1.15	0.001	0.18	0.0029
Odoribacter	1.734	0.0013	0.24	0.0037
Syntrophomonas	2.622	0.0017	0.3	0.0046
Neisseria	-5.043	0.0017	0.32	0.0047
Unclassified.Bacteroidales	1.442	0.0023	0.41	0.0061
Caloramator	1.715	0.0026	0.47	0.0068
Alloiococcus	-1.518	0.0026	0.47	0.0068
Helicobacter	-3.416	0.0031	0.57	0.008
Actinobaculum	-7.919	0.0034	0.62	0.0086
Sedimentibacter	1.655	0.0035	0.64	0.0087
Ignatzschineria	-14.77	0.0036	0.64	0.0087
Pantoea	2.364	0.0039	0.71	0.0094
Unclassified.Solibacterales	-6.776	0.005	0.91	0.012
Collinsella	1.413	0.0051	0.92	0.012
Adlercreutzia	-4.107	0.0073	1	0.017
Unclassified.Veillonellaceae	1.702	0.008	1	0.018
Varibaculum	1.724	0.0083	1	0.019
Phascolarctobacterium	1.498	0.01	1	0.023
Faecalibacterium	1.302	0.012	1	0.026
Arthrobacter	-4.483	0.012	1	0.027
Mycoplasma	2.988	0.015	1	0.032
Sphingobium	-3.533	0.019	1	0.04
Kocuria	2.79	0.021	1	0.043
Unclassified.Rikenellaceae	-1.203	0.022	1	0.046
Roseburia	-1.083	0.025	1	0.052
Unclassified.Lactobacillaceae	-3.374	0.027	1	0.055
Pelosinus	-1.596	0.03	1	0.061
AF12	1.225	0.036	1	0.072
Acinetobacter	-3.148	0.038	1	0.075
Geobacter	1.062	0.045	1	0.087
Unclassified.Neisseriaceae	-1.398	0.047	1	0.09
Paracoccus	-1.053	0.054	1	0.1
Enterococcus	-1.179	0.066	1	0.13
Leptotrichia	-3.279	0.076	1	0.14
Parabacteroides	-1.922	0.081	1	0.15
Sutterella	-5.098	0.088	1	0.16
Unclassified.Paenibacillaceae	-1.196	0.11	1	0.19
Symbiobacterium	-4.816	0.11	1	0.2

Staphylococcus	-1.869	0.12	1	0.21
Enhydrobacter	-2.418	0.12	1	0.21
Chryseobacterium	-2.675	0.12	1	0.22
Sphingomonas	-1.643	0.13	1	0.23
Haemophilus	-2.023	0.13	1	0.22
Wolbachia	-2.859	0.14	1	0.23
Aggregatibacter	1.088	0.14	1	0.24
Oscillospira	-1.383	0.15	1	0.25
Phyllobacterium	-1.332	0.16	1	0.26
Mycobacterium	-1.512	0.16	1	0.26
Rothia	-2.088	0.17	1	0.27
Pelotomaculum	-1.032	0.17	1	0.27
Unclassified.Streptophyta	-3.172	0.18	1	0.28
Dialister	1.108	0.19	1	0.29
Desulfosporosinus	-1.338	0.19	1	0.29
Chroococcidiopsis	12.31	0.2	1	0.31
Unclassified.RF39	-1.258	0.21	1	0.32
Carnobacterium	-1.16	0.21	1	0.32
Tepidimicrobium	1.105	0.22	1	0.33
Lautropia	-9.06	0.23	1	0.34
Achromobacter	-1.664	0.23	1	0.34
Unclassified.YS2	-3.569	0.25	1	0.36
Unclassified.Gemellaceae	-2.135	0.25	1	0.36
Capnocytophaga	-1.13	0.25	1	0.36
Tissierella_Soehngenia	-1.76	0.28	1	0.41
Bilophila	-1.311	0.29	1	0.42
Unclassified.Erysipelotrichaceae	1.091	0.34	1	0.48
Methylobacterium	-1.533	0.34	1	0.48
Bifidobacterium	-1.596	0.34	1	0.48
Bacteroides	-1.707	0.35	1	0.49
Unclassified.OPB54	-5.676	0.36	1	0.49
Burkholderia	-2.341	0.37	1	0.51
Lachnospira	-1.483	0.38	1	0.51
Facklamia	-1.657	0.39	1	0.52
Desulfovibrio	-1.809	0.39	1	0.52
Unclassified.MLE112	-3.853	0.4	1	0.53
WAL_1855D	-2.038	0.41	1	0.54
Fusobacterium	-1.756	0.41	1	0.54
Megasphaera	-2.338	0.43	1	0.56
Blautia	-1.234	0.43	1	0.56

Campylobacter	-1.373	0.44	1	0.56
Dermabacter	-4.032	0.45	1	0.57
Proteus	-1.954	0.47	1	0.59
Acidaminococcus	-1.064	0.49	1	0.61
Micrococcus	-2.801	0.52	1	0.65
Sneathia	-2.198	0.56	1	0.69
rc44	1.815	0.57	1	0.7
Veillonella	-1.787	0.59	1	0.72
Turicibacter	-2	0.6	1	0.72
Peptostreptococcus	-2.727	0.6	1	0.72
Dermacoccus	-2.61	0.6	1	0.72
Stenotrophomonas	-2.086	0.63	1	0.74
Unclassified.S247	-1.464	0.65	1	0.76
Unclassified.Bacillales	-1.809	0.66	1	0.76
Finegoldia	-3.337	0.66	1	0.76
Eikenella	-1.672	0.66	1	0.76
Candidatus_Arthromitus	-2.061	0.68	1	0.78
Janthinobacterium	-1.921	0.69	1	0.79
Moraxella	-1.631	0.71	1	0.8
Unclassified.ML615J28	-1.871	0.72	1	0.8
SMB53	-2.048	0.72	1	0.8
Dehalobacterium	-2.649	0.73	1	0.81
Prevotella	-1.739	0.75	1	0.83
Brachybacterium	-2.333	0.76	1	0.84
Peptoniphilus	-2.596	0.78	1	0.85
Anaerostipes	1.011	0.81	1	0.88
Unclassified.03196G20	-4.599	0.82	1	0.88
Unclassified.RF32	-1.961	0.85	1	0.9
Caldicoprobacter	-2.043	0.85	1	0.9
Shuttleworthia	-2.217	0.86	1	0.9
Pseudoxanthomonas	-3.164	0.86	1	0.9
Actinomyces	-1.658	0.86	1	0.9
Selenomonas	-3.757	0.89	1	0.93
Comamonas	-2.271	0.9	1	0.93
Porphyromonas	-1.242	0.94	1	0.97
Acetobacter	-2.159	0.95	1	0.98
Brevibacillus	-2.066	0.96	1	0.98
Atopobium	-1.961	0.97	1	0.98
